# PACA nanoparticles target and deliver sildenafil to rejuvenate aged mouse liver sinusoidal endothelial cells

**DOI:** 10.7150/ntno.103000

**Published:** 2025-05-14

**Authors:** Tetyana Voloshyna, Christopher Holte, Jakub Pospíšil, Karolina Szafranska, Ole Martin Fuskevåg, Sabina P. Strand, Andreas K.O. Åslund, Yrr Mørch, Sofie Snipstad, Einar Sulheim, Nicholas J. Hunt, Victoria C. Cogger, David G. Le Couteur, Erik Sveberg Dietrichs, Peter A.G. McCourt

**Affiliations:** 1 Vascular Biology Research Group, Dept. Medical Biology, University of Tromsø UiT The Arctic University of Norway, Tromsø, Norway; 2 Experimental and Clinical Pharmacology Research Group, Dept. Medical Biology, University of Tromsø UiT The Arctic University of Norway, Tromsø, Norway; 3 Division of Biotechnology and Nanomedicine, SINTEF, Sem Sælands vei 2 A, Trondheim, Norway; 4 NADENO NANOSCIENCE AS, Dybdahls veg 5, 7051 TRONDHEIM, Norway; 5 Department of Physics, Norwegian University of Science and Technology, Trondheim, Norway; 6 Cancer Clinic, St. Olav's Hospital, Trondheim, Norway; 7 Institute of Research in Biomedicine, Università della Swizzera italiana, Bellinzona, Switzerland; 8 ANZAC Research Institute, University of Sydney, Australia; 9 Department of Oral Biology, University of Oslo, Oslo, Norway

**Keywords:** nanoparticle, targeted delivery, PACA, liver sinusoidal endothelial cell, LSEC, sildenafil, ageing

## Abstract

Ageing is established as the most significant risk factor for disease. About 75% of people over 75 years have diabetes or pre-diabetes and/or hyperlipidaemia which are established risk factors for cardiovascular outcomes, and risk factors for age-related conditions such as dementia, sarcopenia, frailty and osteoporosis. Age-related changes in the liver microcirculation, in particular relating to the cells lining the blood vessels, the liver sinusoidal endothelial cells (LSEC), are a potential cause for dyslipidaemia and insulin resistance in old age. There is also loss of LSEC mediated waste clearance functions essential for homeostasis. Finding ways to reverse these age-related changes in the LSEC will fill a significant gap in therapeutic options available for the treatment of ageing disorders. Such therapies may also benefit patients with fibrotic livers, since LSEC changes in this disease resemble those seen in the ageing LSEC in many aspects. Nanoparticles that access systemic circulation frequently accumulate in the liver. This could be utilized as a promising strategy for targeted drug delivery to the liver. The present study assessed if poly(alkyl-cyanoacrylate) nanoparticles (PACA NPs) are a suitable vector for the targeted transport of such therapeutics to LSEC, to reverse age-related changes such as fenestration/porosity loss. Mice were co-injected with PACA NPs and formaldehyde denatured serum albumin (FSA) and their livers were then examined by microscopy. PACA and FSA co-localised to LSEC, including at the sub-cellular level in endocytic vesicles. Isolated LSEC were challenged with Nile Red (NR668) labelled PACA NPs, which were rapidly internalized. HEK293 cells overexpressing stabilin-2 internalized PACA NPs, suggesting that stabilin-2 mediates PACA uptake on LSEC. Cultured LSEC from aged mice were challenged with PACA NPs containing sildenafil and examined with scanning electron microscopy to determine effects on fenestrations. Sildenafil PACA reversed age-related changes LSEC fenestration frequency and porosity at 3-fold lower sildenafil concentrations than sildenafil alone. If sildenafil PACA induces similar changes *in vivo*, age-related reduction of LSEC porosity could be reversed by the targeted delivery of sildenafil via PACA NPs.

## Introduction

Ageing is a significant risk factor for many diseases and organ dysfunction, including the liver 1. Importantly, the endothelium of the liver is significantly affected both by ageing and pathological processes in disease. In healthy individuals, liver sinusoidal endothelial cell (LSEC) fenestrations facilitate bi-directional exchange between the plasma in the sinusoidal lumen and the hepatocytes in the parenchyma, which is necessary for both drug and metabolite exchange and transformation 2, 3. The LSEC microvasculature is perforated by numerous patent transcellular nanopores without diaphragms or underlying basement membrane, termed fenestrations. Fenestrations are dynamic structures regulated by the Rho kinase (ROCK) and myosin light chain (MLC) kinase pathways 4. They cover 2-20% of the LSEC surface area and this area fraction is defined as 'LSEC porosity' 2, 3, 5. During the ageing process 6-11 and in liver diseases such as metabolic dysfunction-associated steatotic liver disease (MASLD) or cirrhosis 12, 13 the LSEC lose the majority of their fenestrations leading to inefficient bi-directional transport, dyslipidaemia and insulin resistance 14, 15. Specifically, in old age, impaired hepatic clearance of chylomicrons is associated with hypertriglyceridemia and age-related hyperlipidaemia 15, 16. This loss of LSEC fenestrations with ageing is termed either “defenestration” or “pseudocapillarization” 10. In ageing this process is accompanied by expression changes in various proteins, such as ICAM-1, laminin, caveolin-1, and collagens 10, as well as in a decrease in the capacity for waste macromolecule endocytosis, the other main function of LSEC 17, 18.

Several drugs such as sildenafil, simvastatin, metformin, 2, 5-dimethoxy-4-iodoamphetamine (DOI) and xanthines (such as caffeine and theobromine) have been shown to modulate size and number of fenestrations in LSEC 19-23, offering the potential to ameliorate defenestration and related effects. This offers potential to counteract the negative impact of some drugs, ageing and liver disease on LSEC function 19. Sildenafil, and other phosphodiesterase inhibitors have shown promise in increasing LSEC porosity 20. However, both sildenafil and other potential re-fenestrating compounds have potentially serious side effects 24. To avoid such adverse effects from high systemic doses, nanoparticle-based delivery systems have been proposed to deliver a smaller dose in a targeted manner to the LSEC 25, 26.

The intrinsic scavenging activity of LSEC offers a pathway to target them therapeutically, as these cells are the major site for uptake of many nanoparticles (NPs) 17, 27. Indeed, the liver is the main actor in the removal of nanomedicines, and therefore also a potential therapeutic target 28. The receptors stabilin-1 and -2 are highly expressed by LSEC 29-31 and appear to be the main mediators of such uptake 32. In zebrafish, liposomes are taken up via stabilin-2 and small anionic NPs via stabilin-1 32. Targeted delivery through the mannose receptor (CD206) also potentiates delivery to LSEC, due to their greater endocytic capacity relative to other cells 17.

NPs targeting LSEC with re-fenestrating agents have shown promising effects *in vitro* and *in vivo* 22. This had a positive effect on liver fibrosis in an *in vivo* mouse model 22, where the NPs allowed access of therapeutic drugs and appeared to reduce the contribution of LSEC to the inflammatory state.

We propose using poly(alkyl-cyanoacrylate) (PACA) NPs, namely the subtype poly(2-etylbutyl cyanoacrylate) (PEBCA) NPs to deliver therapeutic re-fenestrating agents directly to the sinusoidal endothelium. It was previously reported that PACA NPs accumulated in the liver and spleen, when administered intravenously 33. This suggests that the NPs are potentially taken up via the scavenger receptors stabilin-1 and -2, which are abundant in both organs 29, 30, 33. Therefore, PACA NPs are a potential vehicle for targeting LSEC and carrying compounds to increase their porosity. In the present study we determined the hepatocellular fate of PACA NPs, and their utility in delivering re-fenestrating therapeutics directly to LSEC *in vitro*.

## Materials and Methods

### Reagents

Fibronectin was isolated from human plasma using Gelatin-Sepharose 4B (Cat. No. 17-0956-01, GE Healthcare, Sydney, Australia) according to the manufacturer's instructions. The following reagents were obtained from Sigma-Aldrich, St. Louis, US/Mannheim, Germany: bovine serum albumin, heat shock fraction, pH 7, ≥98% (Cat. No. A7906); Liberase™ TM Research grade (Cat. No. 5401127001); sodium cacodylate trihydrate ≥98% (Cat. No. C025); RPMI1640 (Cat. No. R0883); Penicillin-Streptomycin (Cat. No. 4333) ethanol, pure, anhydrous ≥99.5% (Cat. No. 459836); hexamethyldisilazane (HMDS) reagent grade, ≥99% (Cat. No. 440191). For MACS cell isolation, the following reagents were obtained from Miltenyi Biotec, Bergisch Gladbach, Germany: CD-146 (LSEC) MicroBeads mouse (Cat. No. 130-092-007); autoMACS rinsing solution (Cat. No. 130-091-222); MACS BSA Stock Solution (Cat. No. 130-091-376); MS Columns (Cat. No. 130-042-201); pre-separation filters (70 µm) (Cat. No. 130-095-823). Osmium tetroxide 4%, aqueous solution (DG) (Cat. No. C011) was obtained from ProSciTech, Thuringowa, Australia. Paraformaldehyde 16% solution, EM Grade (Cat. No. 15710) was obtained from Electron Microscopy Sciences, Hatfield, USA. Glutaraldehyde 25% solution, EM Grade (Cat. No. 16210) was obtained from Laborimpex, Brussel, Belgium. CultureWell™ Removable Chambered Coverglass (Cat. No. CS16) was obtained from Grace Biolabs, Bend, Oregon, US. LDH-Glo™ Cytotoxicity Assay (Cat. No. J2380) was obtained from Promega, Madison, USA. alamarBlue™ Cell Viability Reagent (Cat. No. DAL1025) was obtained from Thermo Fisher Scientific, Eugene, USA. PACA reagents: 2-EBCA (Cuantum Medical Cosmetics, Barcelona, Spain), Miglyol 812 (IOI Oleo), Brij L23 (Cat. No. P1254, Sigma-Aldrich), Pluronic F-127 (Cat. No. P2443, Sigma-Aldrich), Kolliphor HS-15 (Cat. No. 4296, Sigma-Aldrich), docusate sodium (Cat. No. D-1685, Sigma-Aldrich), 3M HCl in methanol (Cat. No. 90964, Supelco).

### PACA nanoparticles

PACA NPs (a PEBCA-subtype) were synthesized using a mini-emulsion polymerization method as previously described 33. The sildenafil was encapsulated in the form of docusate salt. First, sodium docusate was converted into docusic acid by addition of 3M methanolic HCl to the sodium docusate solution in chloroform at the molar ratio 1:1. The precipitated NaCl was removed by filtration (0.45 µm PVDF filter) and chloroform evaporated. Then, sildenafil-docusate was prepared by mixing molar equivalent amounts of sildenafil (free base) and docusic acid in chloroform. The solvent was evaporated, and the resulting sildenafil-docusate salt used for encapsulation. To prepare vector control (VC) PACA NPs, sildenafil-docusate was replaced by Miglyol 812. PACA NPs used in the LSEC uptake experiments were labelled by lipophilic dye NR668 in the oil phase (0.25% w/w, synthesized in our lab according to Klymchenko et al. (2012) 34. After emulsification, PACA NPs were left to polymerize for 48 h at room temperature. The resulting particles were characterised by dynamic- and electrophoretic light scattering on Zetasizer Nano ZS (Malvern Panalytical, UK) to provide size, size distribution and zeta potential. The sizes are reported as Z-average of hydrodynamic diameter calculated by instrument software from cumulant analysis. Zeta potentials were calculated from electrophoretic mobilities measured in disposable capillary cells using general purpose mode. All measurements were performed in 10 mM phosphate buffer pH 7.4.

*Sildenafil release from PACA NPs:* The release of sildenafil from PACA NPs was measured using a microdialysis plate with 10 kDa cut-off (Pierce, 88260). Briefly, particles were diluted in TRIS-buffered saline pH 7.6 containing 0.05% Tween 20 (Sigma-Aldrich, 91414) to an effective sildenafil concentration of 0.8 mg/mL. The particle suspension (three replicate samples of 0.1 mL each) was loaded into the chamber of the dialysis device and placed into a 96-well plate filled with 1.8 mL of TRIS-buffered saline with 0.05% Tween 20. The particles were dialysed for 24 h at 37 °C, and the samples of the dialysate were sampled at regular intervals (5 min, 10 min, 15 min, 30 min, 60 min, 120 min, 260 min and 24 h). The sildenafil content in the dialysates was analysed by LC-MS/MS as described below.

*Sildenafil quantification: S*ildenafil loading in PACA NPs was assessed by LC-MS/MS and found to be 6-8% of total dry weight. A 6-point inhouse calibration curve was prepared in acetonitrile ranging from 0.01 to 1,000 nM for sildenafil (Sigma-Aldrich, Schnelldorf, Germany). Sildenafil-d3 (Toronto Research chemicals, Toronto, Canada) was used as internal standard (IS). Analysis was performed on a Waters Acquity* I*-class UPLC with an autosampler, and a binary solvent manager connected to a Waters Xevo TQ-XS benchtop tandem quadrupole mass spectrometer with an UniSpray™ (US) ion source (Waters, Manchester, UK). Chromatography was performed on a Cortecs T3, 120Å, 1.6 µm, 2.1 mm × 100 mm (Waters) column maintained at 50 °C. A linear gradient composed of (A) 0.1% aq formic acid and (B) acetonitrile with 0.1% formic acid was used. The gradient was as follows: 0: 10% B; 0-2 min, 70% B; 2-2.5 min, 80% B; 2.51-3.5, 10% B. The autosampler temperature was 4 °C and the sample injection volume was 1 μL. The mass spectrometer was operated in positive UniSpray mode (US+) and impactor voltage was set to 0.90 kV. The system was controlled by MassLynx version 4.2 software. Desolvation gas temperature was 500 °C; source temperature was 150 °C; desolvation gas flow was 1,000 L/h; cone gas flow. The following multiple reaction monitoring (MRM) transitions were used; *m/z* 475->58/**100** (sildenafil)**,** *m/z* 478->61/**103** (sildenafil-d3) where bold number is qualifier.

By dividing the amount of sildenafil as determined by MS with the total dry weight of the respective sildenafil PACA preparation loaded in the MS we calculated the percentage of the sildenafil cargo, and used this to calculate the effective sildenafil concentrations used in cell challenge studies. The formula used was as follows: (MS determined mass of sildenafil) / (total mass of sildenafil PACA assayed by MS) x 100 = % sildenafil cargo in sildenafil PACA NP.

### Characterization of PACA by transmission electron microscopy

Sildenafil PACA (Pill 81 in Table 1) was diluted 1:100 in double distilled H_2_O, and pipetted (5 µL) onto a 400 mesh Cu-grid with carbon coated formvar film. The sample was quickly washed with double distilled H_2_O (4-5 drops) followed by incubation with 1% uranyl acetate for 1 min. Excess uranyl acetate was removed using blotting paper and sample then dried prior to imaging on a Hitachi HT7800 120 kV transmission electron microscope.

### *In vivo* experiments

**Figure 1:**
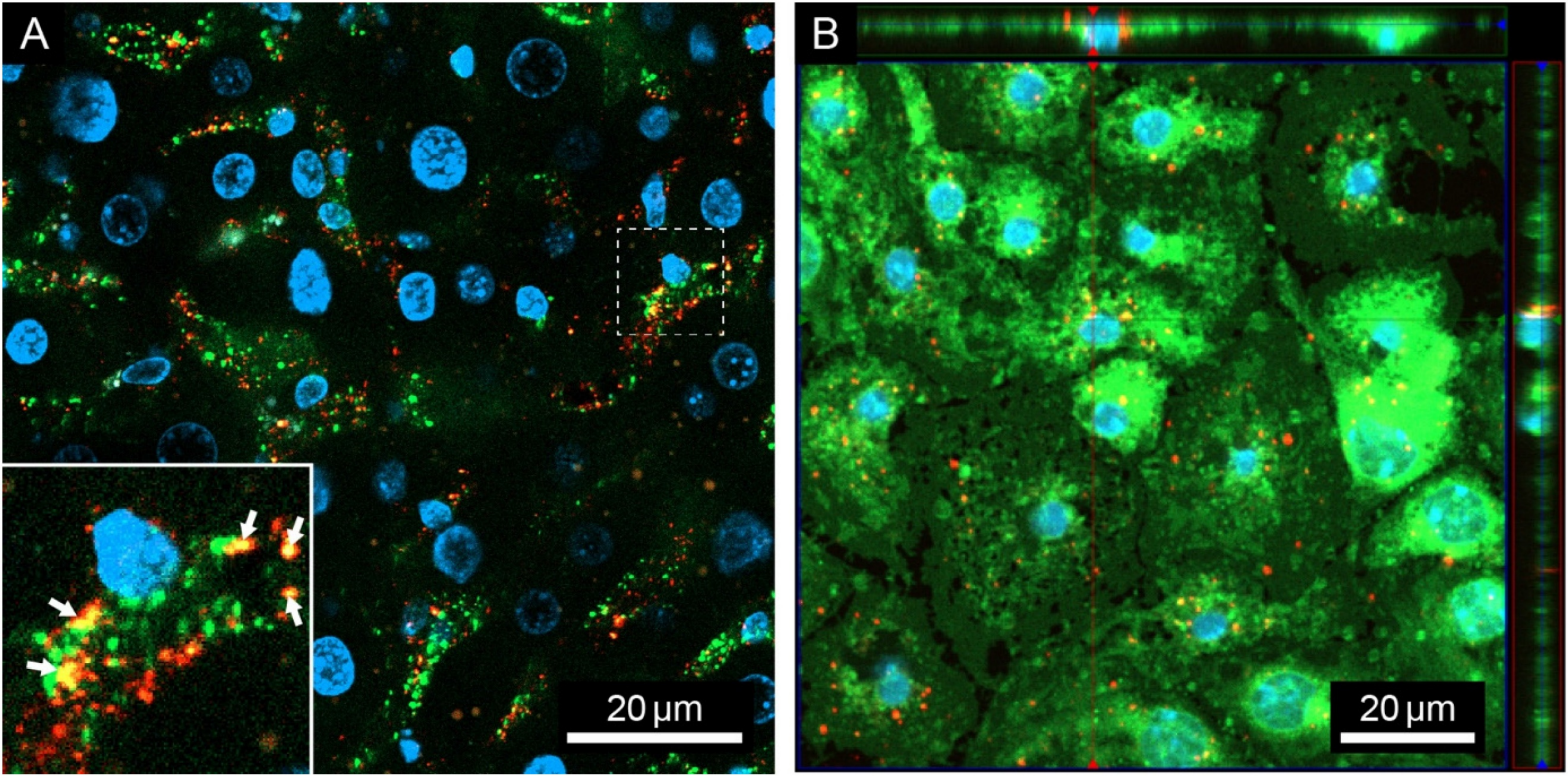
Uptake of PACA NPs loaded with NR668 dye (PACA-NR668) in mouse liver sinusoidal endothelial cells (LSEC): (A) *in vivo* in a liver section and (B) *in vitro* in cultured mouse LSEC. (A) Liver section from one mouse injected with formaldehyde treated serum albumin-AlexaFluor488 (FSA-AF488) (2.5 mg/kg) and PACA-NR668 (225 mg/kg). Liver sinusoids appear diffuse green, indicating uptake of FSA-AF488, arrows in inset indicate colocalization (yellow) of PACA-NR668 (red) and FSA-AF488 (green) in LSEC when administered *in vivo*. The sample was stained with DAPI (blue). Selected cross-section shows uptake of PACA-NR668 and its intracellular perinuclear localisation. (B) Uptake of PACA-NR668 (red) in cultured (young) mouse LSEC (n = 1). Localization of PACA-NR668 indicated in z-stack (red arrows). The sample was stained with CellMask™ Green (green) and DAPI (blue).

Female Balb/c nude mice (8-12 weeks old) were housed in groups of 5-6 in a specific pathogen free environment, at the Comparative Medicine core facility at NTNU. The animals were kept in autoclaved individually ventilated cages with 65 air changes per h at 23 °C, 50-65% relative humidity and a 12 h light/dark cycle, with free access to food and sterile water, and cages were enriched with bedding, housing, nesting material and gnaw sticks. The mice were anesthetized using isoflurane. Two mice received formaldehyde treated serum albumin-AlexaFluor488 (FSA-AF488) in PBS (2.5 mg/kg) and PACA NPs loaded with NR668 dye (BC-30 in Table 1) in PBS (4.5 mg/20 g mouse, injected in a 50 mL volume, corresponding to a dose of 225 mg/kg) injected intravenously and sequentially in the tail vein. Animal 1 received FSA-AF488 first, then PACA-NR668 5 min later, and was euthanized 10 min after FSA injection (Figure 1A). Animal 2 received the PACA-NR668 first, then FSA-AF488 50 min later, and was euthanized 60 min after PACA injection (Figure S1). The livers were harvested and immediately frozen in liquid nitrogen before 8 µm sections were made. The sections were coverslip mounted with Vectashield/DAPI then imaged on a Leica SP8 confocal microscope. The experimental protocols were approved by the Norwegian Food Safety Authority (Mattilsynet) approval no. 13195.

### *In vitro* experiments

#### Animals

The experimental protocols were approved by the ethics committee of the Sydney Local Health District Animal Welfare Committee (AWC# 2022/025 approval 02/2023-1/2026) and Local Animal Research Authority at the UiT The Arctic University of Norway (09/22). All experiments were performed in accordance with relevant national and local guidelines and regulations and reported in accordance with ARRIVE guidelines. The total number of mice used in this study is 3 young and 14 aged.

Young mice, aged 8-14 weeks, (strain C57 Black6JRj) were purchased from Janvier (Germany/France) and housed at the Department for Comparative Medicine at UiT, at standard conditions, given water and chow diet *ad libitum*.

Aged mice, aged 26-28 months, (strain C57Black6J) were obtained from the Animal Research Centre in Perth, Western Australia. The old mice were housed at the ANZAC Research Institute, Sydney, Australia, on a 12 h light/dark cycle and provided with *ad libitum* access to a standard chow mouse diet, water, and enrichment.

### LSEC isolation and culture

The mice were anesthetized with a mixture of 10 mg/kg xylazine and 100 mg/kg ketamine before sacrifice. LSEC were extracted as described by Elvevold et al. (2022) 35. Briefly, livers were perfused via the portal vein and digested with Liberase™ TM Research grade (Sigma-Aldrich, St. Louis, US, 1.2 mg in 50 mL isotonic sodium/potassium HEPES buffer pH 7.4). Hepatocyte and non-parenchymal cell (NPC) fractions were separated by differential centrifugation (35 x g for 2 min at 4 °C; max acceleration/deceleration). The supernatant containing the NPC fraction was then centrifuged at 300 x g for 10 min at 4 °C; max acceleration/deceleration, and pellet resuspended in 150 mL freshly made rinsing buffer (MACS BSA Stock Solution diluted 1:20 with autoMACS Rinsing Solution). LSEC were isolated from the NPC fraction by immuno-magnetic cell separation, selecting for CD-146 (Miltenyi, MACS). Mouse CD-146 MicroBeads were added to the cell suspension in a 1:20 ratio and incubated for 30 min on a rotating mixer at 4 °C. Cells were then washed and centrifuged at 300 x g for 10 min at 4 °C and resuspended in rinsing buffer. The cell suspension was then added to a pre-rinsed LS Column with a 70 mm cell strainer and washed with rinsing buffer (3 x 3 mL). The column was removed from the magnetic separator and magnetically labelled LSEC were flushed into a new collection tube. Cells were counted and seeded on human fibronectin coated wells (10 min 0.2 mg/mL of human fibronectin).

LSEC were cultured in serum free RPMI 1640 supplemented with 10,000 U/mL penicillin, 10 mg/mL streptomycin (Cat. no. P4333, Sigma-Aldrich) at 37 °C in 5% CO_2_ and 20% O_2_. The cells were incubated for 1 h to allow attach to the substrate. Media was then exchanged to remove non-viable cells, and the cells were further incubated for 1-2 h prior to treatments. Seeding information is described for each assay.

### Uptake of PACA nanoparticles in cultured mouse LSEC

Young mouse LSEC were seeded on 0.2 mg/mL fibronectin coated ø13 mm round glass coverslips and challenged with 20 µg/mL of vector control PACA-NR668 in RPMI 1640 for 30 min. Cells were washed 3 times with preheated PBS prior to fixation with 4% formaldehyde in PBS for 15 min. Samples were stained with 1:1000 CellMask Green (Thermo Fisher Scientific) in PBS for 30 min and mounted on glass slides in VectaShield with DAPI. Images of the samples were taken using confocal microscopy (Zeiss LSM800) a processed using ZEN software (Zeiss).

### Uptake in HEK293 cells expressing stabilin-1 and -2

As a model for stabilin-mediated uptake in LSEC, an established model using HEK293 cells expressing mouse stabilin-1 or -2 36, or the empty plasmid vector pEF6V5His-TOPO (Merck) were used. The HEK-cells were cultured in low glucose DMEM (Sigma-Aldrich) supplemented with 7% FBS (Biowest/Sigma-Aldrich), 10,000 U/mL penicillin, 10 mg/mL streptomycin and 10 µg/mL blasticidin (Merck), as described by Hansen et al. (2005) 36.

The cells were seeded on 0.2 mg/mL fibronectin coated plastic culture wells and challenged with 20 µg/mL PACA-NR668, either in the presence or absence of unlabelled FSA (200 µg/mL) alone, or in the presence of both FSA-AF488 (20 µg/mL) and unlabelled FSA (200 µg/mL). The cells were incubated for 90 min in phenol red free RPMI 1640 containing 1% human serum albumin (HSA).

### LSEC morphology studied with scanning electron microscopy

Aged mouse LSEC were seeded at a density of 1.8×10^5^ cells/cm^2^ on fibronectin coated chambered coverglass (CS16-CultureWell™ Removable Chambered Coverglass, Grace Bio-labs, OR, USA) and treated with matched concentrations (0-0.1-1-5-10-25-50-75 µg/mL) of sildenafil PACA or vector control PACA NPs. Working solutions of PACA NPs were freshly prepared in PBS, while all treatments were prepared in serum free RPMI 1640. The cells were incubated with treatments for 1 h, then washed with preheated PBS and fixed with 2.5% glutaraldehyde and 4% formaldehyde solution in PBS.

The sample preparation method for scanning electron microscopy (SEM) was as follows; the cells were post-fixed for 1 h with freshly made and filtered (0.2 µm) 1% tannic acid in 0.1 M sodium cacodylate buffer, then 1 h in 1% OsO_4_ in 0.1 M sodium cacodylate buffer and dehydrated with a graded series of ethanol (30%, 60%, 90%, and 4 x 100% for 5 min) before chemical drying with HMDS (2 x 2 min). The specimens were sputter coated with 10 nm Au/Pd and scanned using the Zeiss Gemini 300 scanning electron microscope.

Cells were chosen at random from three or more different regions of the well. PACA NPs concentrations at 5 µg/mL and above appeared to be toxic for LSEC in the *in vitro* context, causing cell detachment. Thus, only lower concentrations (0.1 and 1 µg/mL) were examined further. A total of 32-46 images from a total of 3 biological replicates were analysed per treatment group.

Fenestrations were counted manually in the whole cell, and the fenestration area was calculated using improved version of semi-automated segmentation method based on the Fiji thresholding 37. The key difference between the Fiji thresholding method and the approach used in this work lies in preprocessing the input data. Before thresholding, each SEM image was denoised using a convolutional neural network (DnCNN) 38. This preprocessing step improved the segmentation of fenestrations in SEM images with lower quality where the semi-automatic method failed. In the final step of segmenting the fenestrations, the false detections were excluded based on the locations marked during the manual counting.

Assuming the fenestrations are elliptical, maximum diameter 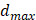
and minimum diameter 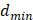
were automatically measured for each fenestration. Using these measurements, the area of each fenestration was calculated separately as the area of an ellipse. By summing these individual areas, we obtained the total fenestrated area of each cell. Dividing this value by the cell area gives the cell porosity 37. Also, equivalent circle diameter 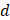
was determined as 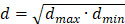, representing the fenestration diameter as a single value, which simplifies comparisons and analyses.

### Viability and toxicity assays

For alamarBlue cell viability assay, LSEC from aged mice were seeded on fibronectin coated 96-well plate at a cell density of 1.5×10^5^ cells/cm^2^ and treated with matched concentrations (0-0.1-1-5-10-25-50-75 µg/mL) of sildenafil PACA and vector control PACA NPs. All experiments included cell-free control wells. The cells (and cell-free wells) were incubated with 10% alamarBlue (Thermofisher Scientific, Eugene, USA) solution in culture media for 3 h following a 1 h long pre-treatment with sildenafil- and vector control PACA NPs prior to readout. The assay was repeated in 3 biological replicates using 3 batches of PACA NPs.

The lactate dehydrogenase (LDH) release cytotoxicity assay was performed using commercial luminescence LDH detection kit (LDH-Glo™, Promega) to quantify lactate dehydrogenase release upon plasma membrane disruption. LSEC from aged mice were seeded on standard 48-wellplates (1.8-2.8×10^5^ cells/cm^2^) and treated with matched concentrations (0-0.1-1-5-10-20-50-75 µg/mL) of sildenafil PACA or vector control PACA NPs. Wells without cells served as negative controls to determine cell media background. After 1 h, a 25 µL aliquot of the cell culture media was collected into 225 µL of freezing buffer (details in the manufacturer's protocol) and stored at -20 °C until analysis. The standard manufacturer's protocol was followed. The assay was repeated in 4 biological replicates and 3 batches of PACA NPs.

### Statistics

#### Fenestration frequency and porosity, alamarBlue and LDH measurements

All fenestration/porosity scores (within the same experimental group) from 3 biological replicates were pooled, giving 32-46 images per experimental group. Statistical analyses were carried out using Prism 10 (Graphpad Software). Normal distribution of the data was not confirmed after assessment by D'Agostino & Pearson, Anderson-Darling, Shapiro-Wilk and Kolmogorov-Smirnov tests. Differences in fenestration frequency and porosity between equal concentration-treatment with 0.1 and 1 µg/mL of sildenafil PACA and vector control PACA NPs, as well as within-group differences of either sildenafil PACA or vector control PACA NPs and differences between the untreated control group with every other group, were assessed by Kruskal-Wallis test and thereafter compared using Dunn's multiple comparisons test.

The differences in the LDH data were assessed by Kruskal-Wallis test as normality was not confirmed. Within-group differences of either sildenafil PACA or vector control PACA were compared by Dunn's multiple comparisons test, where all concentrations (0.1-75 µg/mL) were compared to the untreated control group.

Normal distribution of the alamarBlue data was assessed, and confirmed, by D'Agostino & Pearson, Anderson-Darling, Shapiro-Wilk and Kolmogorov-Smirnov tests. Within-group differences between treatments (0.1-75 µg/mL) of either sildenafil PACA or vector control PACA were assessed by ordinary one-way ANOVA, and each treatment group (0.1-75 µg/mL) was thereafter compared to untreated control group by Dunnett's multiple comparisons test.

P-values < 0.05 were considered statistically significant.

## Results

### Characterization of PACA nanoparticles

The characteristics of the PACA particles used in this study are given in Table 1. All particles had hydrodynamic diameters below 200 nm and polydispersity index close to or below 0.2. The sildenafil loading was approximately 6-8% w/w of particle dry weight.

The release profile of sildenafil from PACA NPs in Tris-buffered saline pH 7.6 containing 0.05% Tween-20 at 37 °C showed a cumulative release of 5% after 2.5 h and 15% after 24 h (Figure S5).

Sildenafil PACA NPs were characterized by transmission electron microscopy (Figure S6) which revealed polydiverse spheres of 50-200 nm diameter.

### *In vivo* experiments

To determine the hepatocellular distribution of i.v. injected PACA NPs, mice were injected sequentially with FSA-AF488 (a ligand specifically taken up by LSEC) (2.5 mg/kg) and then with PACA-NR668 (4.5 mg/20 g mouse, injected in a 50 mL volume, corresponding to 225 mg/kg), or *vice versa*. The purpose of the sequential administration was to avoid the risk of one reagent binding to the other pre-injection. Figure 1A shows a liver section from an animal that received FSA-AF488 first, then PACA-NR668 5 min later, and was sacrificed after another 5 min. The LSEC in the section can be identified by diffuse green staining from FSA-AF488 uptake in the liver sinusoids, as well as intracellular green vesicular staining. Within the LSEC, red staining indicates the uptake of PACA-NR668 in the vesicles. Co-localisation of FSA-AF488 and PACA-NR668 in the same vesicles, resulting in a yellow colour, is indicated by the arrowheads in Figure 1A. The injection of both ligands in the reverse order in another mouse (Figure S1) revealed similar results.

### *In vitro* experiments

#### LSEC uptake

To determine the fate of PACA NPs in LSEC *in vitro*, cultured LSEC from young mice were challenged with PACA-NR668 (20 µg/mL). Due to the rapid endocytic capacity of LSEC, the cells were also treated with monensin to arrest the PACA-NR668 and prevent its degradation in lysosomes. Figure 1B shows the peri-nuclear accumulation of PACA-NR668 in intracellular vesicles (arrows) which is typical for material endocytosed by LSEC.

#### HEK293 uptake

**Figure 2:**
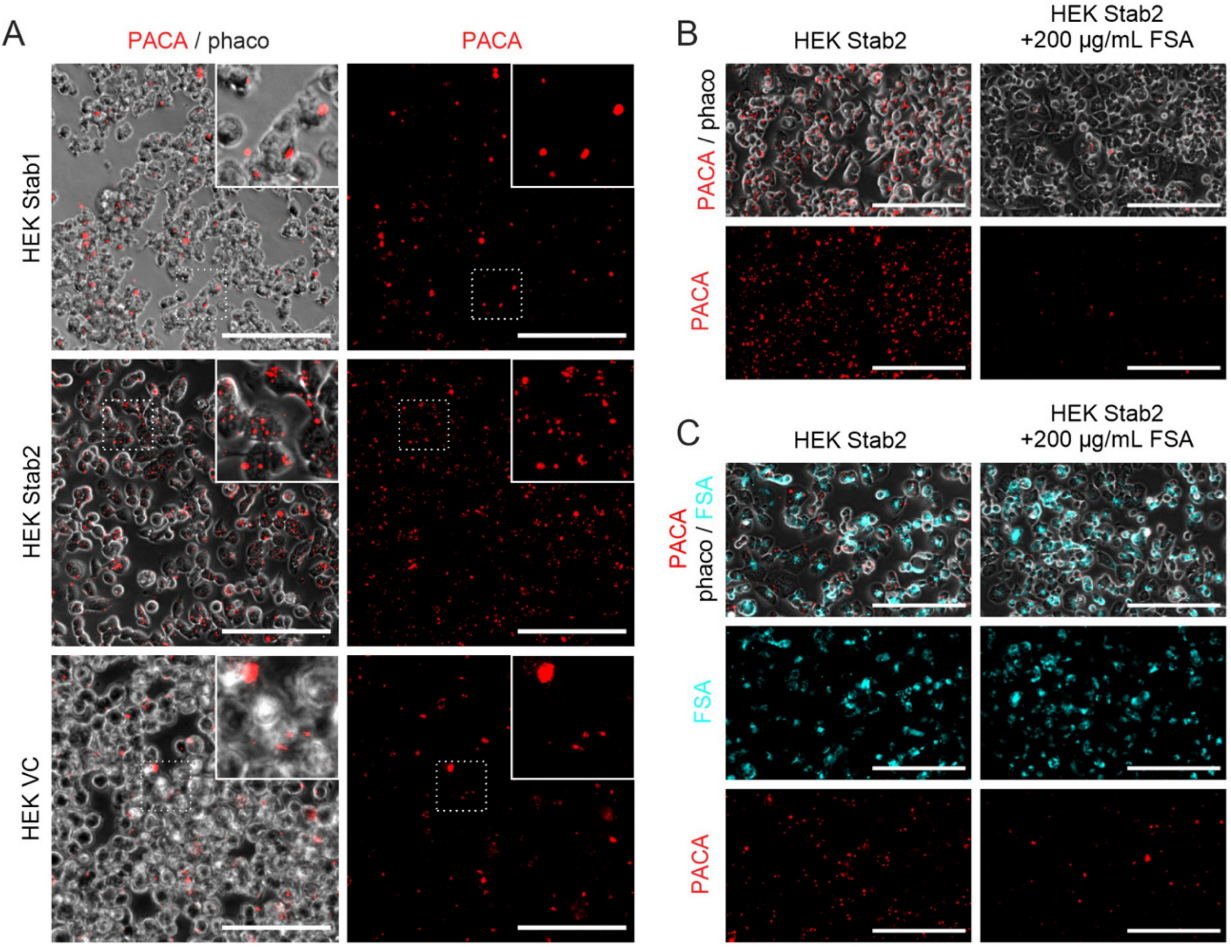
Stabilin-2 in HEK cells enables more uptake of PACA NPs than stabilin-1. HEK293 cells were treated with PACA-NR668 (20 µg/mL) for 90 min, in serum (5% FBS). (A) Uptake of PACA-NR668 NPs in stabilin-1, stabilin-2 or vector-plasmid control transfected HEK293 cells. Left panels: merge of phase-contrast and red channel; right panels: NR668 channel alone. Stab1 = stabilin-1 transfected HEK293 cells, Stab2 = stabilin-2 transfected HEK293 cells, VC = vector-plasmid control transfected HEK293 cells. (B) Inhibition of PACA nanoparticle uptake by the stabilin-1/2 ligand FSA (200 µg/mL, unlabelled) in HEK293 cells expressing stabilin-2. Left panels: PACA uptake in the absence of inhibiting concentration of FSA (200 µg/mL, unlabelled), Right panels: PACA uptake in the presence of inhibiting concentration of FSA (200 µg/mL, unlabelled). (C) Uptake of PACA nanoparticle co-incubated with FSA-AF488 (20 µg/mL) ± unlabelled FSA (200 µg/mL). PACA = PACA NPs, phaco. = phase contrast, FSA-AF488 = AlexaFluor488 labelled FSA. Top left and right panels: merged FSA-AF488, PACA-NR668 and phaco channels; middle left and right panels: FSA-AF488 channel alone; bottom left and right panels PACA-NR668 channel alone. All scale bars are 100 µm.

To ascertain the roles of stabilin-1 and -2 in the uptake of PACA NPs, HEK293 cells over-expressing each stabilin were used as a model for stabilin uptake in LSEC and challenged with PACA-NR668 (Figure 2A). HEK cells expressing murine stabilin-2 took up PACA-NR668 while HEK cells expressing stabilin-1 or transfected with the empty plasmid vector showed minimal uptake (Figure 2A).

The inhibition of PACA NP uptake with FSA, a stabilin-1/2 ligand, in HEK expressing stabilin-2 is shown in Figure 2B. The addition of 200 µg/mL FSA to the culture media markedly reduced the uptake of PACA (Figure 2B, right panels).

Figure 2C shows HEK stabilin-2 cells co-incubated with PACA (20 µg/mL) and either FSA-AF488 (20 µg/mL) alone (left panels), or FSA-AF488 (20 µg/mL) and FSA (200 µg/mL) (right panels). The top two panels in Figure 2C show merged AF488, NR668 and phase contrast channels, while the panels below show the AF488 (middle left and right) and the NR668 channels (bottom left and right) alone, respectively. FSA-AF488 also inhibits the uptake of PACA, as seen all 3 left panels of Figure 2C. As a control, FSA (200 µg/mL) also reduced the uptake of AF488-FSA (Figure 2C right panels) and further reduced the uptake of PACA.

#### Effect of PACA nanoparticles on LSEC morphology

The efficacy of PACA NPs as a vector for transport of sildenafil to LSEC was assessed by quantifying the fenestration frequency (number of fenestrations per 1 µm^2^) and porosity (percentage of cell area covered in fenestrations) of cultured LSEC from aged mice (Table 2). The cells were challenged with fresh cell culture media, vector control PACA NPs, and sildenafil PACA NPs for 1 h.

**Figure 3:**
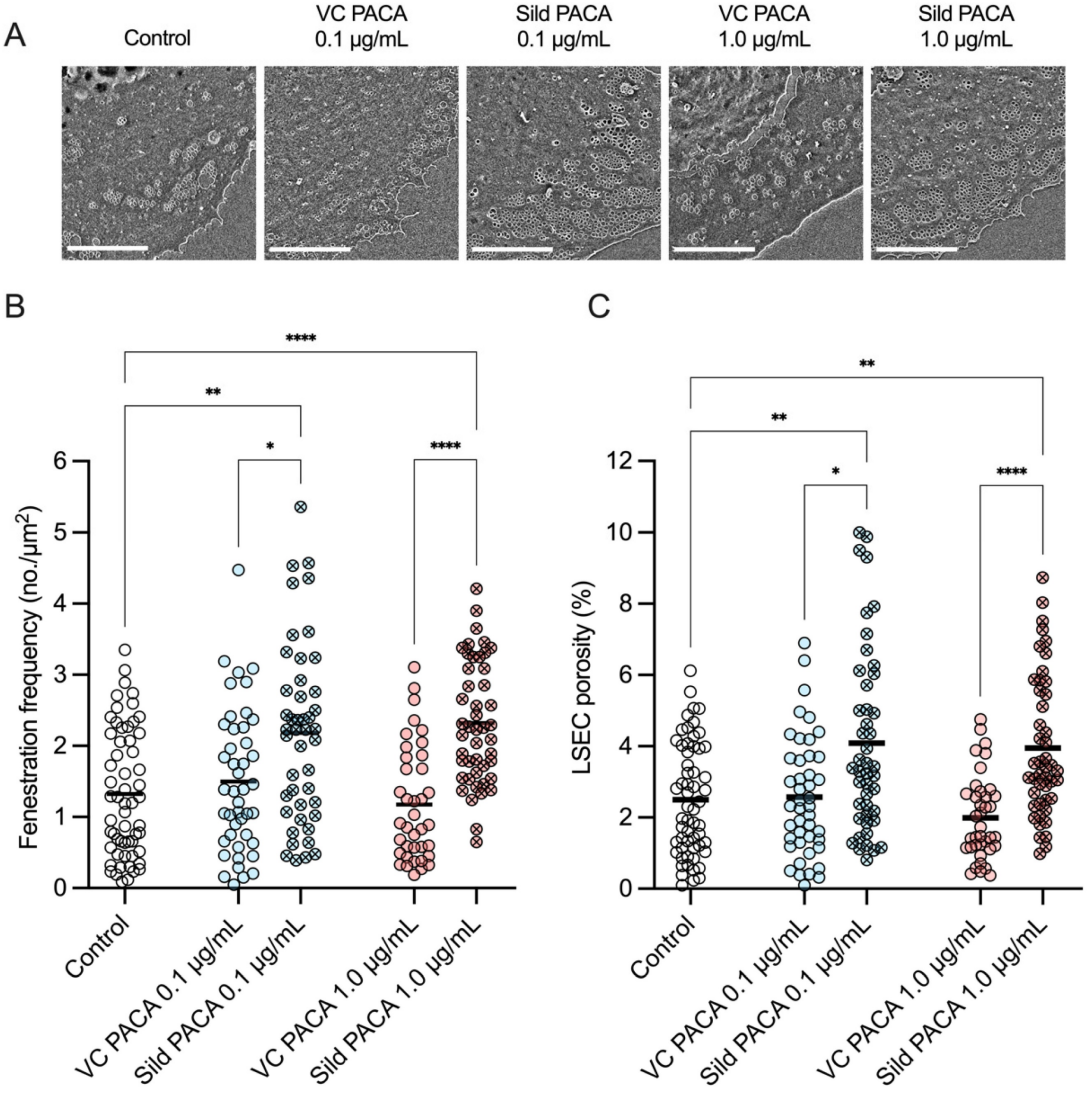
Representative images (A) of LSEC from aged mice in the various treatment groups. Effect of PACA NPs on (B) fenestration frequency (number of fenestrations/µm^2^) and (C) porosity (percentage of cell surface covered by fenestrations) of cultured LSEC. LSEC were challenged with either control cell media, vector control PACA or sildenafil PACA NPs for 1 h. Fenestration frequency (B) and porosity (C) are increased in cells treated with 0.1 µg/mL and 1 µg/mL sildenafil PACA NPs, compared to the untreated control and the matched concentrations of vector control PACA NPs. There is no significant difference in fenestration frequency or porosity between the untreated control cells and cells treated with vector control PACA. The data is presented as individual points representing single cells from 3 biological replicates (4 for the untreated control group), with a line representing the mean, and is based on 8⁠-21 images per biological replicate per treatment group. *, ** and **** indicate statistically significant differences (p<0.05; p<0.002; p<0.0001, respectively) between the sildenafil PACA and untreated control/vector control PACA groups. VC PACA = vector control PACA, Sild PACA = sildenafil PACA. All scale bars are 5 µm.

An increase in fenestration frequency was observed in cells treated with 0.1 and 1 µg/mL sildenafil PACA NPs, both compared to untreated control cells and cells treated with matched concentrations of vector control PACA (Figure 3A). A similar trend was also observed when assessing the change in LSEC porosity (Figure 3B). The drug loading of sildenafil PACA (Pill 18 in Table 1) was measured to be 7.6% of the total content in the stock (see table 1). With this drug loading, the effective sildenafil concentration at PACA concentrations 0.1 and 1 µg/mL corresponds to ≈ 7.6 and ≈ 76 ng/mL, respectively. To determine if similar equivalent concentrations of a standard dose of non-encapsulated sildenafil had any effect on LSEC porosity, LSEC from aged mice were challenged with 60 ng/mL sildenafil. No effects were seen at this sildenafil concentration (Fig S3).

**Figure 4:**
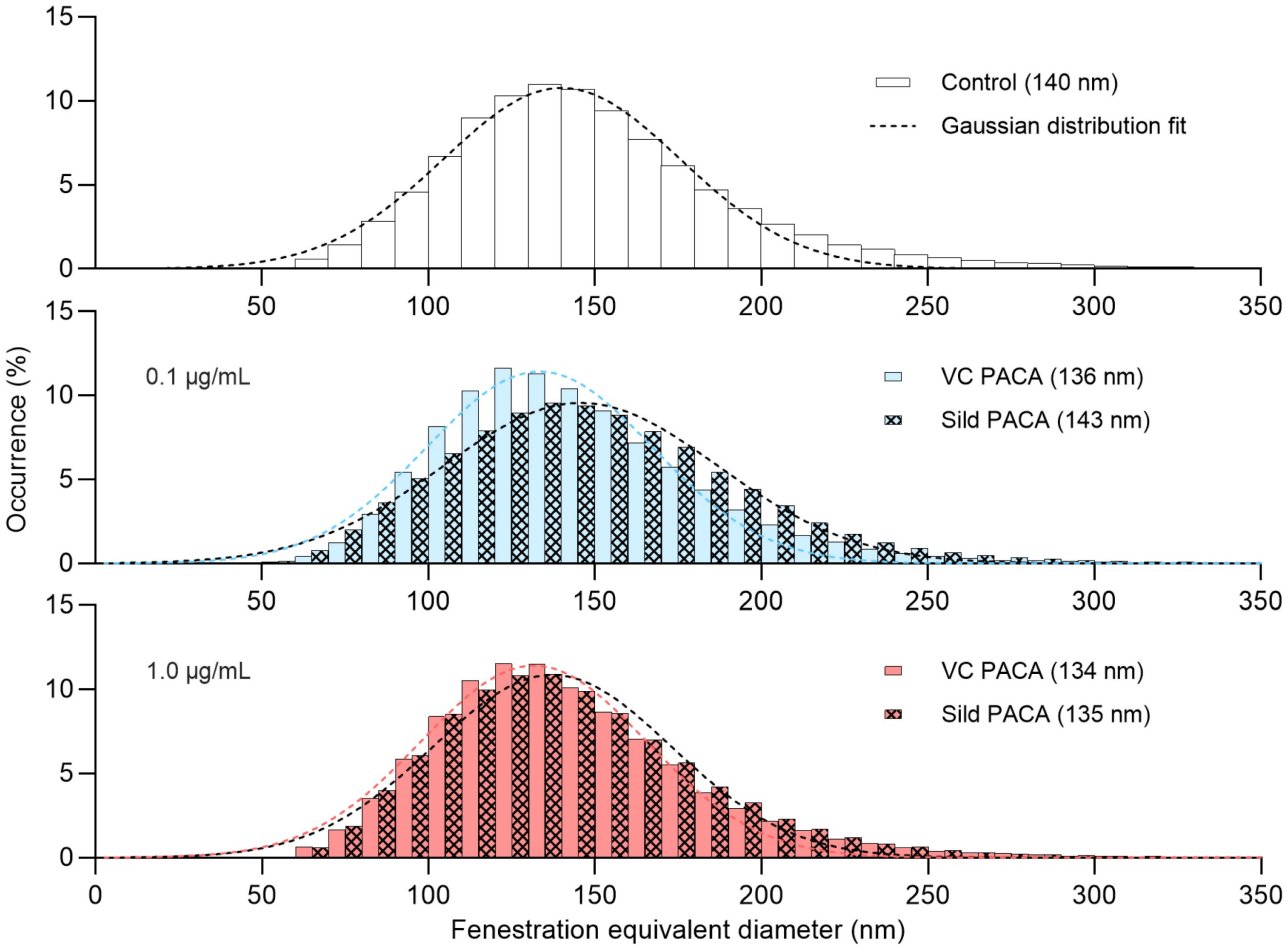
Distribution of the fenestration diameter of liver sinusoidal endothelial cell (LSEC) isolated from aged mice. Each histogram represents equivalent diameter measurements of fenestrations from 3 biological replicates (4 for the untreated control group), with 8-21 images per replicate for each treatment group. First row shows untreated control group, followed by 0.1 µg/mL (blue) and 1 µg/mL (red) of vector control PACA (plain color) and sildenafil PACA (oblique square pattern). The dashed lines represent fitted Gaussian curves. The centers of each Gaussian distribution are noted in the legend for each treatment group separately.

There was no significant difference in fenestration diameter between cells in the untreated control group and cells treated with either sildenafil PACA or vector control PACA NPs (Figure 4).

#### Viability and cytotoxicity assay

**Figure 5:**
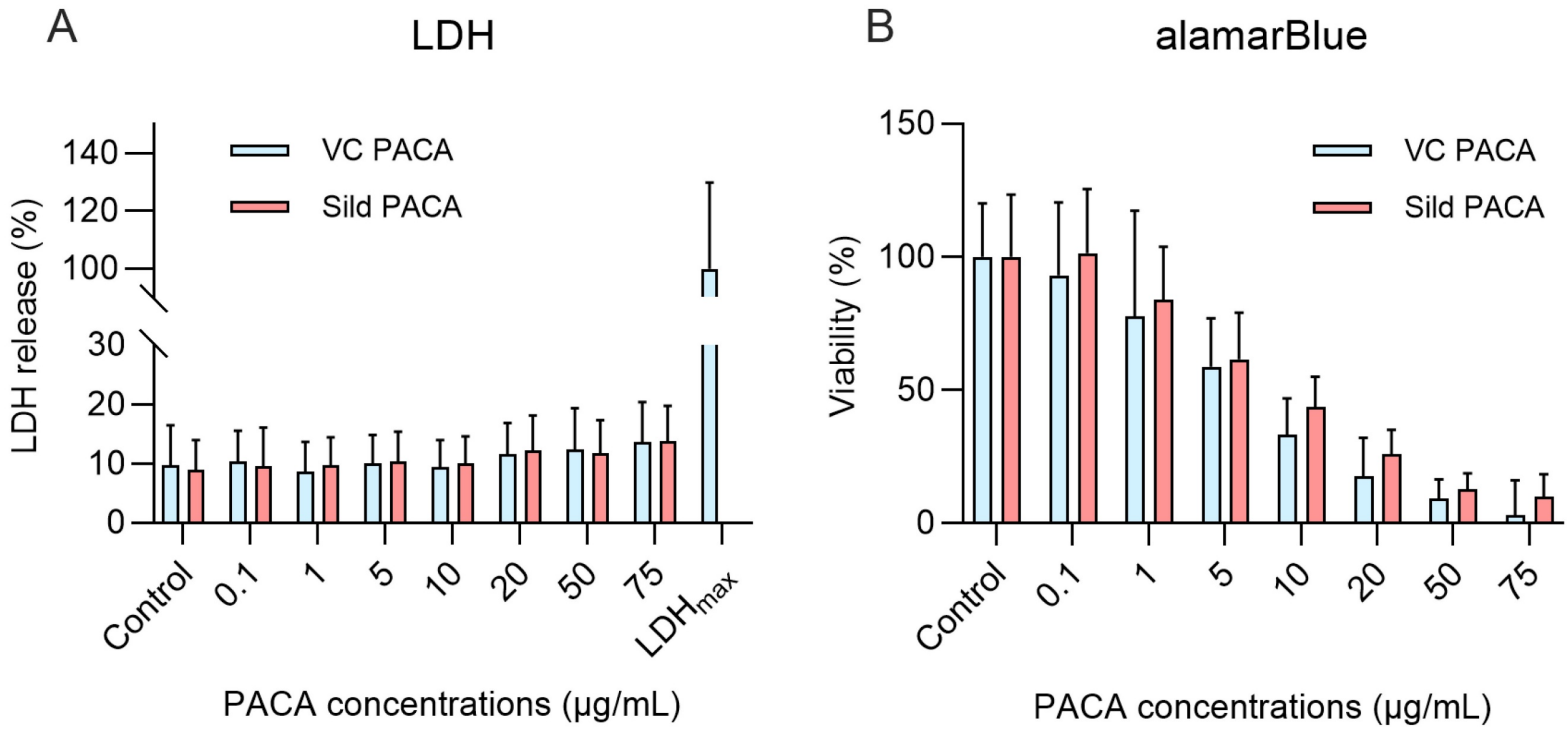
Viability and toxicity assays on cultured liver sinusoidal endothelial cells (LSEC) from aged mice challenged with control media, and with matching concentrations of sildenafil-loaded- and vector control PACA NPs. Lactose dehydrogenase cytotoxicity assay (LDH) (A) shows the effect on LSEC following 1 h challenge with PACA NPs. LDH data is based on 4 biological replicates and presented as means with standard deviations. There is no significant difference in LDH release between PACA-treated groups and the untreated control. For the alamarBlue cell viability assay (B), LSEC were pre-treated with PACA NPs for 1 h followed by a 3 h incubation with 10% alamarBlue reagent. There was no significant difference in cell viability between the untreated control group and 0.1 and 1 µg/mL PACA NPs, but ≥5 µg/mL of both sildenafil PACA and vector control PACA NPs significantly reduces cell viability. The alamarBlue data is based on 3 biological replicates and presented as means with standard deviation.

Two viability/cytotoxicity assays were performed to examine the toxic effects of PACA NPs on cultured LSEC isolated from aged mice; the lactate dehydrogenase release cytotoxicity assay (LDH) to assess cell death upon loss of cell membrane integrity, and the alamarBlue assay to assess the change in reducing power of the cells and thereby functional cell viability. The cells were treated with fresh cell culture media, vector control PACA NPs, or sildenafil PACA NPs. 1 h exposure of LSEC to sildenafil-loaded and vector control PACA NPs did not increase the LDH release significantly compared to the untreated control group (Figure 5A). For the alamarBlue assay, the cells were pre-treated with PACA NPs for 1 h, followed by 3 h incubation with 10% alamarBlue prior to readout. A 4 h (Figure 5B) or 16 h (supplementary Figure S4) exposure of LSEC to 0.1 and 1 µg/mL PACA NPs did not significantly decrease cell viability compared to the untreated control group. However, 5 µg/mL and above decreased the reducing power of the cells significantly compared to the untreated control group (Figure 5B).

## Discussion

During ageing, there is a significant loss of LSEC fenestrations and thereby LSEC porosity, resulting in reduced communication and transfer of substrates between hepatocytes and the blood plasma 39. However, fenestrations are dynamic structures and are responsive to a variety of pharmacological interventions 19. Several pharmaceuticals, including sildenafil, have previously been shown to increase the number of fenestrations and porosity in LSEC, also when isolated from aged mice 20. While these pharmaceuticals have the potential to prevent or reverse defenestration and the associated effects, unwanted side effects are common at high systemic doses 24, 40. By incorporating the re-fenestrating compounds into nanoparticle-based drug delivery systems targeting LSEC, such as PACA NPs, we can potentially reduce systemic side effects while lowering the drug dosage.

### PACA nanoparticle administration and delivery

Our results demonstrate the efficacy of PACA mediated delivery of sildenafil for the purposes of improving LSEC function. Although we show that PACA NPs accumulate in LSEC following intravenous administration, other methods of administration are also possible and have been explored, including intraperitoneal, subcutaneous and intramuscular administration 41-43. Oral delivery remains the preferred and most attractive route of administration due to its simplicity and conveniency 44, 45, however, the majority of nanomedicines on the market today are injectables 33, 46. Nevertheless, Bagad and Khan (2015) 47 and Kafka et al. (2011) 48 showed that PACA-like nanoparticles seem to survive the gastrointestinal tract following oral administration and increase the bioavailability of the cargo. To achieve effective targeting to LSEC, the entire particle would have to cross the gastrointestinal barriers and enter the circulation as an intact particle 44, 49, 50. Whether this is possible with the current PACA NPs remains to be examined. Hunt et al. (2020) 21 achieved LSEC targeting using orally administered Ag_2_S based quantum dots (QDs) coupled with bound materials, showing a rapid intestinal uptake and targeted delivery to LSEC *in vivo* following oral administration. Moreover, coating the QDs with FSA increased their uptake in LSEC by 3-fold, while a gelatin biopolymer coating increased the uptake by hepatocytes 21. Similarly, metformin and nicotinamide mononucleotide (NMN) conjugated to QDs had greater bioavailability and selective accumulation in the liver following oral administration in mice, compared to unconjugated compounds 22. More recently, Hunt et al. (2024) 51 reported that insulin coupled to Ag_2_S QDs and encapsulated in a chitin-glucose shell survived oral administration and reached the liver where insulin exerted its effect in rodents and non-human primates. Further studies with novel coatings on PACA nanoparticles could potentially overcome the issue of the gastrointestinal barrier.

### PACA nanoparticle uptake

Co-administration of FITC-FSA and vector control PACA NPs into mice resulted in detection of both materials in the sinusoids and within intracellular vesicles of the LSEC, with evidence of colocalization of FITC-FSA and PACA in the same vesicles. This demonstrates that FSA and PACA can be taken up by the same endocytic route, most likely via the endocytosis receptors stabilin-1 and stabilin-2, which bind FSA 36. *In vitro* challenge of LSEC with vector control PACA NPs revealed that PACA was taken up in endocytic vesicles. Some of these localized to perinuclear regions, which is consistent with LSEC endocytic fate of collagen alpha chains and stabilin-1/-2 ligands 36, 52. Sulheim et al. (2016) 53 demonstrated efficient uptake and colocalization of PACA NPs with early endosomes, late endosomes and lysosomes in rat brain endothelial cell line RBE4 and showed that clathrin-mediated endocytosis was the dominating pathway, although caveolae-mediated endocytosis also contributed to the uptake.

PACA NPs challenge of HEK cells overexpressing stabilin-2 demonstrated uptake of PACA NPs. Stabilin-1 HEK cells and HEK transfected with empty plasmid demonstrated some limited uptake, but considerably less than the stabilin-2 expressing cells. Ligands specific for stabilin-1 and stabilin-2 inhibited the uptake in stabilin-2 overexpressing HEK cells. This would suggest that both stabilin-2, and to a lesser degree stabilin-1, are involved in uptake of PACA NPs, which is consistent with zebrafish studies by Campbell et al. (2018) where liposomal NPs were taken up by stabilin-2, but not stabilin-1 32.

### LSEC morphological effects

The treatment of isolated LSEC from aged mice with 0.1 and 1 µg/mL sildenafil PACA demonstrated a 1.5-2-fold increase in fenestration frequency and porosity compared to both the untreated control- and the vector control PACA group. Previous studies by Hunt et al. (2018) 20 showed a 2-fold increase in fenestration frequency and porosity in LSEC cultured from aged mice treated with 300 and 600 ng/mL free sildenafil. The effective doses of sildenafil (i.e. the cargo within the PACAs) used in our study (7.6 and 76 ng/mL) were 4-40 times lower than the lowest sildenafil concentration (300 ng/mL) used in Hunt et al. (2018) 20. In addition, we show that a nearly equivalent dose (60 ng/mL) of free (non-encapsulated) sildenafil had no effect on LSEC porosity (Figure S3), confirming that the PACA vector enhances the effect of sildenafil. Consistent with Hunt et al. (2018) 20, we did not see any increase in fenestration diameter in LSEC treated with sildenafil PACA NPs.

Our findings therefore indicate that sildenafil is released from PACA NPs inside the cell upon uptake where it exerts its effect. Moreover, we show that PACA NPs potentiate the effect of sildenafil. This potentiating effect could suggest more effective targeting of PACA to LSEC as well as more efficient uptake via scavenger receptors (stabilin-1/2) compared to that of free sildenafil. A similar potentiating effect was shown by Hunt et al. (2021) 22, where metformin and nicotinamide mononucleotide (NMN) conjugated to Ag_2_S QDs increased physiological, metabolic, and cellular potency compared to unconjugated formulations by 25- and 100-fold, respectively.

In the endothelium, sildenafil acts by increasing intracellular cGMP by selective inhibition of phosphodiesterase (PDE) 5 54, 55. Interestingly, in a transcriptomic and proteomic study of rat liver cells by Bandari et al. 56, it was reported that PDE-5 is close to absent in LSEC. The mechanism by which sildenafil exerts its effect on LSEC therefore remains to be elucidated.

### Viability

The viability and toxicity assays performed showed that lower doses (0.1 and 1 µg/mL) of PACA NPs are well tolerated by isolated LSEC. The LDH assay suggested that cells were intact at all concentrations of PACA, however, when observing the cells in SEM, 5 µg/mL PACA NPs and above caused apparent defenestration when compared to control cells, indicating toxicity, while concentrations above 20 µg/mL disrupted the cell membrane the cells, suggesting apoptotic rather than necrotic cell death. The functional viability assay alamarBlue revealed PACA associated toxicity at 5 µg/mL and above. These results are consistent with the toxicity profiles of PACA NPs, particularly of PEBCA, in the Hep G2 and LLC-PK1 cell lines demonstrated by Sulheim et al. (2017) 57. Nevertheless, doses of 0.1 and 1 µg/mL were well tolerated *in vitro* according to both assays, and we also demonstrated that therapeutic effects of sildenafil PACA occur at lower concentrations than the toxic doses of this pharmaceutical.

The average drug loading for PACA NPs is around 10% for hydrophobic drugs 58. As of now, a maximum of ~ 100 ng/mL sildenafil can be delivered to cultured LSEC before reaching PACA associated toxicity. Liu et al. (2020) 59 demonstrated an exceptionally high drug loading of 58.5% by using a sequential nanoprecipitation method to encapsulate various hydrophobic drugs, which also resulted in improved therapeutic effect and safety. Higher drug loading could enable the use of lower NP concentrations, thus reduce PACA-associated toxicity, while maintaining the re-fenestrating effect of sildenafil.

Although higher concentrations of PACA (5 µg/mL and above) were not well tolerated in cultured LSEC, previous studies have shown that animals tolerate much higher *in vivo* doses, up to 2 mg per animal (200 mg/kg) 60 which corresponds to a blood concentration of ≈ 4 mg/mL, and as demonstrated by *in vivo* experiments in the present paper, up to 225 mg/kg. This might eliminate the need of increased drug load to achieve the desired therapeutic effects on LSEC *in vivo.* The physiological environment of LSEC *in vitro* differs significantly from that of isolated LSEC. The absence of flow in *in vitro* studies might cause NPs to sediment, potentially increasing their interaction with LSEC. This might result in higher effective concentrations, and subsequently, elevated toxicity levels.

### PACA toxicity profile

The toxicity profile of PACA NP is complex and differs significantly from the toxicity profile of their corresponding cyanoacrylate monomers 57, 61-63. Several studies have reported PACA nanoparticles to be biodegradable or biocompatible 42, 64-66. The first toxicological data on PACA NPs did not demonstrate any acute toxicity, neither at the cellular level, nor at the *in vivo* level 42. Sulheim et al. (2017) 57 published a comprehensive study on the cytotoxicity of PACA NP where the toxicity of 19 different PACA NP batches was evaluated using 12 different cell lines. These authors showed that PACA NPs with intermediate degradation rates, specifically PEBCA NPs, were less toxic than particles with faster or slower rates of degradation. Moreover, neither the particle size, when in the range of 100-200 nm, nor the different combination of polyethylene glycol surfactants seemed to affect the toxicity of PACA NPs significantly. Although PEBCA NP were found to be the least toxic, the toxicity was shown to vary across different cell lines 57.

Fernandes-Urrusuno (1994) 67 studied the toxicological effects of PACA NPs in rats *in vivo/ex vivo*, where PACA nanoparticles were administered intravenously in doses of 20 mg/kg over 14 days, giving a total dose of 200 mg/kg. To evaluate the hepatoxicity of NPs, hepatocytes were isolated and functional and metabolic parameters were measured. Slight alterations in hepatocyte specific functions were found, where an elevation in AGP (a positive acute-phase protein) and a decrease in albumin (negative acute-phase protein) secretion indicated an inflammatory response. Nevertheless, these effects were reversed 15 days after the discontinuation of PACA treatment 67. In contrast, the inflammatory response induced by the non-biodegradable polystyrene nanoparticles was not reversible 67. *In vitro* studies were also performed on hepatocytes and cocultures of hepatocytes and Kupffer cells, where cells were challenged with PACA nanoparticles, or degradation products of PACA particles. No effect was seen in the cocultures or after challenge with degradation products, however challenge of hepatocytes only with PACA NPs resulted in elevated secretion of AGP 67. Thus, the reversibility of toxic effects depends on biodegradability and elimination of nanoparticles.

Recent work by Hyldbakk et al. (2022) 68 described *in vivo* toxicity of PACA NPs, including PEBCA, in healthy rats after intraperitoneal injection. Biochemical parameters showed an acute and transient immune response in rats after a single intraperitoneal injection of either PEBCA or PEHCA NPs (75 mg NPs/kg), nevertheless, all responses were normalized 16 days post injection. Clinical chemistry analysis revealed very few differences between the PACA treated groups and the saline control group. Histopathology showed mild reactions to PACA NPs, however, these were evaluated as effects of local intraperitoneal injections 68. Similarly to the previous study, Hyldbakk et al. (2023) 43 showed that injection of empty NPs into rats (75 mg/kg) and mice (143 mg/kg) induced negligible toxic effects. In the present study, sildenafil PACA at 0.1/1.0 mg/mL increased LSEC porosity *in vitro*. The dose required to achieve these concentrations in a 20 g mouse with a 0.5 mL blood volume would be 2.5/25 mg/kg, which is considerably lower than Hyldbakk et al. (2023) 43 used. The effective *in vivo* concentration of sildenafil PACA remains to be determined, but it is likely to be considerably less than the doses used in Hyldbakk et al. (2023) 43.

### Pharmacokinetic properties of PACAs

Discussions on how size, charge and other physiochemical characteristics affect the biodistribution and pharmacokinetics of NPs has been ongoing for years and is yet to be fully resolved. The choice of monomer and the length of the alkyl chain influences the degradation rate of NPs, moreover, the density of PEG surface coverage and the formation of protein corona are all factors that influence their release kinetics and circulation time 33, 57. Pharmacokinetic parameters of PACA NPs were initially described by Grislain et al. (1983) 42, where blood clearance and excretion, as well as whole-body distribution were determined after intravenous and subcutaneous administration. These authors showed that [C^14^]-labelled PACA NP were rapidly cleared from the blood, with a calculated half-life of 6.63 min. Radioactivity excreted in the urine and feces was measured 1, 3 and 7 days after intravenous administration of the particles, with majority being eliminated on day 7 42. Whole-body autoradiography revealed that most of the radioactivity was localized in the liver (78%), lungs and kidneys 5 min after intravenous injection, however it accumulated mostly in the liver, spleen and bone marrow at later time points. After 24 h most of the radioactivity was cleared 42. On the other hand, subcutaneous and intramuscular administration of [C^14^]-labelled NP showed a different distribution pattern, as most of the radioactivity was concentrated around the site of injection and then in the gut wall, while the whole-body distribution was markedly lower, and no radiation was detected in the liver 42. Biodistribution of PACA, including PEBCA, was recently investigated by Pandya et al. (2022) 60, where the distribution of NR668-labeled PACA NPs was studied by fluorescent imaging up to 48 h after intravenous injection (2 mg in 0.9% NaCl) in mice. PEBCA nanoparticles were shown to accumulate mostly in the liver and spleen, consistent with Grislain et al., but were detectable in most organs including, kidneys, lymph nodes and lungs 60.

More recently, Hyldbakk et al. (2023) 43 described the biodistribution of PACA NPs in mice after either intraperitoneal or intravenous injection (143 mg PACA NPs/kg) using three complementary methods; whole-animal imaging of NR668 labelled PACA, analysis of NP degradation product 2-ethylbutanol (2-EB) and mass spectrometry-based quantification of the PACA-encapsulated cabazitaxel. Whole-animal imaging revealed even distribution of PACA NPs in the peritoneal cavity within an hour after intraperitoneal injection, while after intravenous injection the fluorescent signal from PACA NPs was detected in the entire animal 43. Moreover, analysis of the PACA specific degradation product 2-EB, showed accumulation in the liver and spleen, and was detected as late as 35 days post-injection 43. Also, in tumour bearing mice, 2-EB was shown to accumulate in the liver and spleen, in addition to the tumour, 2 h after intraperitoneal injection 43.

It has previously been suggested that PACA NPs are taken up in the lysosomes and degraded by esterase enzymes 67, 69. The hydrolysis of the lateral ester chain and production of polycyanoacrylic acid and alcohol was identified as the major degradation pathway 69, while formaldehyde and cyanoacetate are minor degradation products 67, 70.

In comparison to free drug, drugs incorporated into PACA NPs were shown to concentrate in the liver and the spleen 42, 43, 67. This biodistribution profile of PACA NPs allowed for treatment of intracellular infections 71-72 and hepatic metastasis 67, 73.

### General discussion

Almost 50% of all Norwegians aged 70-84 years use statins 74 to treat hyperlipidaemia and atherogenesis by reducing cholesterol synthesis and increasing cholesterol uptake in hepatocytes. LSEC function is associated with age-dependent hyperlipidaemia, as LSEC lose their fenestrations with increasing age 10.

These drugs are used to treat hyperlipidaemia and atherogenesis by reducing cholesterol synthesis and increasing cholesterol uptake in hepatocytes. LSEC function is associated with age-dependent hyperlipidaemia, as LSEC increasingly lose their fenestrations with increasing age 10. This leads to impaired hepatic clearance of chylomicrons and is associated with hypertriglyceridemia and age-related hyperlipidaemia 15, 16. The causative role of hyperlipidaemia in development of cardiovascular disease is widely acknowledged and is considered a major modifiable risk factor for preventing such disorders. Despite the high usage of statins, hyperlipidaemia is still underdiagnosed and undertreated in the elderly 75. Hyperlipidaemia-treatment in this group is further complicated by the LSEC-ageing process. Reduced porosity of LSEC gives an unfavourable reduction in statin effect 19, since these drugs are dependent on passing through LSEC-fenestrations to reach the hepatocytes where they inhibit HMG-CoA.

Circulatory disease was the most common cause of death both in men and women aged >65 years in the EU in 2020 76. Pharmacotherapy that potentiates the effect of statins could therefore postpone or prevent the development of one of the most important health-challenges in an ageing population. Sildenafil is a promising candidate for this purpose, as it is known to increase LSEC porosity 20. In the present study we show that sildenafil PACA possess the same ability. This is an important step towards a novel strategy to improve statin effect in the elderly and reduce risk of cardiovascular disease from hyperlipidaemia. Prescribing sildenafil or other phosphodiesterase 5-inhibitors as a standard additional treatment to statins is complicated by their systemic effects. In patients using sildenafil for erectile dysfunction, the most common side effects are headache, dizziness and visual disturbances. Sildenafil is however also used to treat pulmonary hypertension and has systemic vascular effects. Rare adverse events such as lethal cardiac or cerebrovascular events are described during therapeutic use 77, 78. The present study shows the great clinical potential of targeted sildenafil PACA treatment to avoid side-effects from high systemic doses in addition to potentiating the effect of sildenafil. The present results show that this is a promising strategy that could potentiate anti-atherogenic treatment and prevent lethal cardiovascular disease in the elderly. The PACA-sildenafil formulation can therefore become a useful therapy to ameliorate the effects of ageing on the liver and increase the health span of older people.

In conclusion, we show that PACA NPs accumulate in mouse LSEC *in vivo* and are endocytosed by LSEC *in vitro*. PACA NPs are taken up mostly by the receptor stabilin-2, and to a lesser degree stabilin-1 in HEK293 cells expressing these endocytic receptors. Furthermore, PACA NPs potentiate the effect of sildenafil, enabling the use of lower concentrations to achieve the desired therapeutic effect. However, the efficacy of PACA as a vector for LSEC-specific delivery still needs to be demonstrated in *in vivo* studies. Most importantly, we show that PACA NPs are a potentially suitable vector for delivering re-fenestrating pharmaceuticals to LSEC.

## Figures and Tables

**Table 1:** PACA nanoparticle data

Batch name	PACA nanoparticle type	Size (nm)	PDI	Zeta potential (mV)	Total dry content in stock (mg/mL)	Drug loading (% dry weight)
BC-30	PEBCA-NR668	165	0.23	-2.8	-	-
Pill-18	Sildenafil-docusate PEBCA	170	0.136	-2.17	148	7.6
Pill-81	Sildenafil-docusate PEBCA	148	0.17	-2.5	146	6.5
Pill-19	Sildenafil-docusate PEBCA-NR668	180	0.114	-1,56	141	7.5
Pill-17	Vector control PEBCA	169	0.108	-1.15	138	-
Pill-82	Vector control PEBCA	102	0.21	-2.1	145	-
Pill-20	Vector control PEBCA-NR668	173	0.178	-0.584	142	-

**Table 2:** Mean fenestration frequency and porosity of PACA NP treated LSEC from aged mice

Nanoparticle	Control	VC PACA 0.1 µg/mL	Sild PACA 0.1 µg/mL	VC PACA 1.0 µg/mL	Sild PACA 1.0 µg/mL
**Mean fenestration frequency (no./µm^2^)**	1.3 ± 0.6	1.4 ± 0.2	2.1 ± 0.6	1.2 ± 0.1	2.3 ± 0.5
**Mean porosity (%)**	2.5 ± 1.1	2.5 ± 0.3	3.7 ± 1.6	2.0 ± 0.1	3.9 ± 1.6

## Abbreviations

LSEC: liver sinusoidal endothelial cell
PACA: poly(alkyl-cyanoacrylate)
NP: nanoparticle
FSA: formaldehyde treated serum albumin
NR668: Nile Red 668
LC: liquid chromatography
MS/MS: tandem mass spectrometry
AF488: alexa fluor 488
HEK 293: human embryotic kidney cell line
RPMI 1640: Roswell Park Memorial 1640 medium
DAPI: 4′,6-diamidino-2-phenylindole
HSA: human serum albumin
LDH: lactose dehydrogenase
VC: vector control
QD: quantum dot
NMN: nicotinamide mononucleotide
HMG-CoA: 3-hydroxy-3-methylglutaryl coenzyme A
