# Supplementary material for: PACA nanoparticles target and deliver sildenafil to rejuvenate aged mouse liver sinusoidal endothelial cells
Source: Nanotheranostics. 2025 May 14;9(2):155–70. doi: 10.7150/ntno.103000 (PMC12188536; doi:10.7150/ntno.103000)
Supplement: Supplementary file 1 — Supplementary figures and tables. [file ntnov09p0155s1.pdf]

Supplementary information

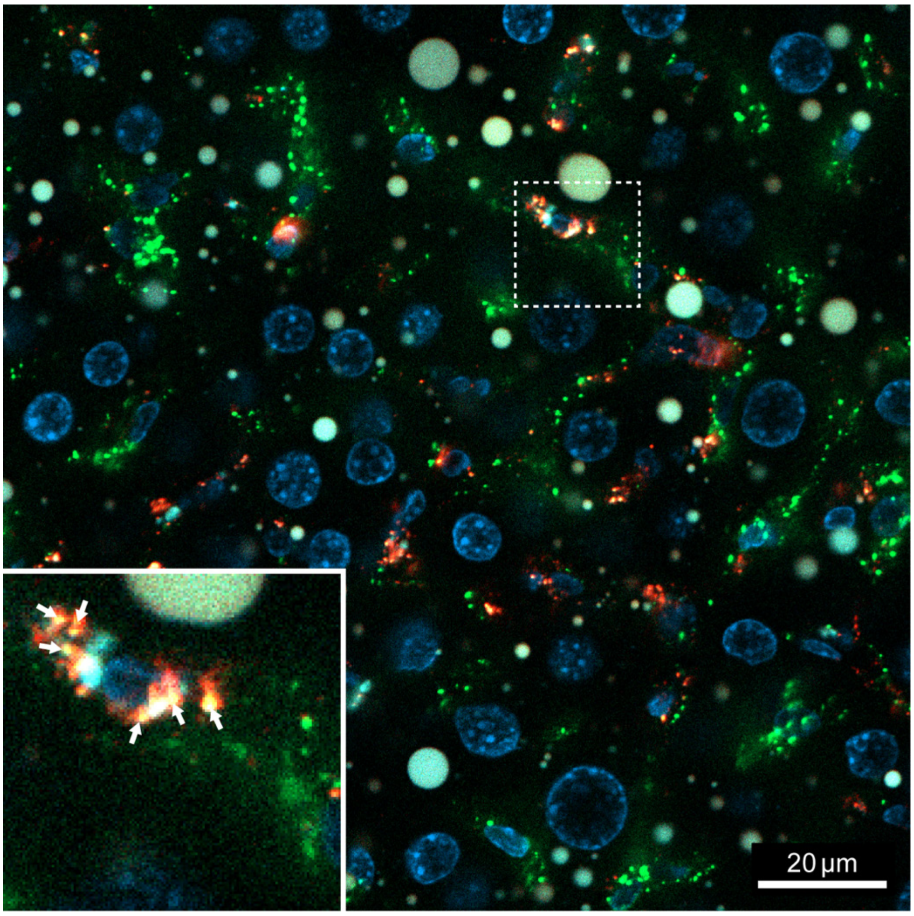

Figure S1. Liver section from one mouse that was injected first with PACA-NR668 (225 mg/kg), then FSA-AF488 (2.5 mg/kg) 50 min later, then euthanized 60 min after the PACA injection. Liver sinusoids appear diffuse green and with green vesicles, indicating uptake of FSA-AF488, arrows in inset indicate colocalization (yellow) of PACA-NR668 (red) and FSA-AF488 (green) in LSEC when administered in vivo. The sample was stained with DAPI (blue). Selected cross-section shows uptake of PACA-NR668 and its intracellular perinuclear localisation.

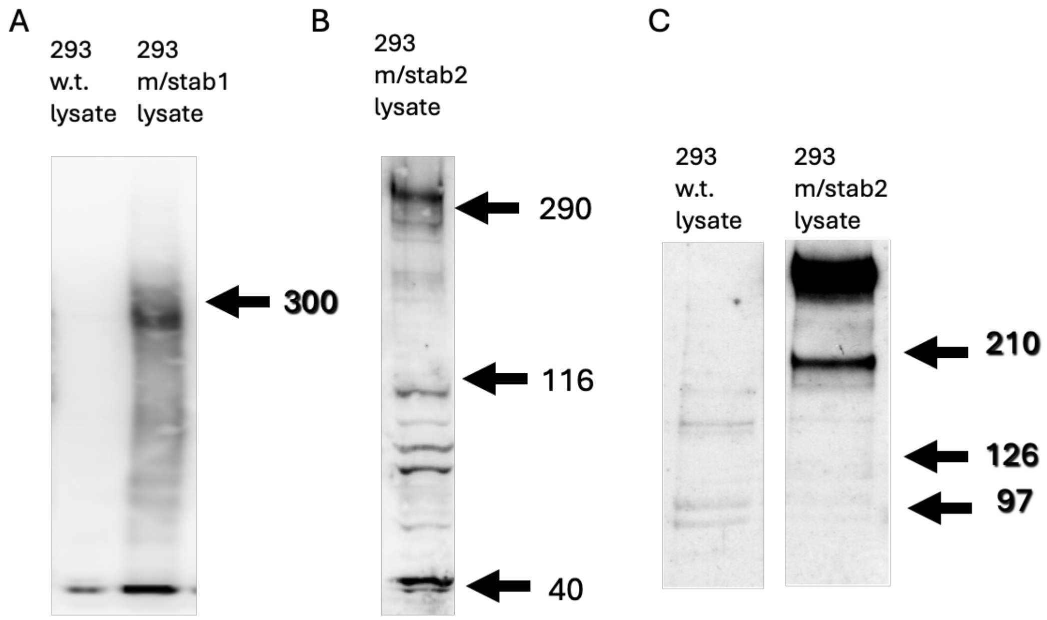

Figure S2. Western blots of lysates from HEK293 cells and HEK293 expressing recombinant mouse stabilin-1 (m/stab1) (A) and mouse stabilin-2 (m/stab2) (B, C), probed with anti-mouse stabilin-1 (Atlas, Cat. No HPA005434) and anti-rat stabilin-2 (McCourt et al. 1999) respectively. The Western blot in C used the same anti-rat stablin-2 antibody and is the original blot from Hansen et al. 2005 [1] and is included for comparison.

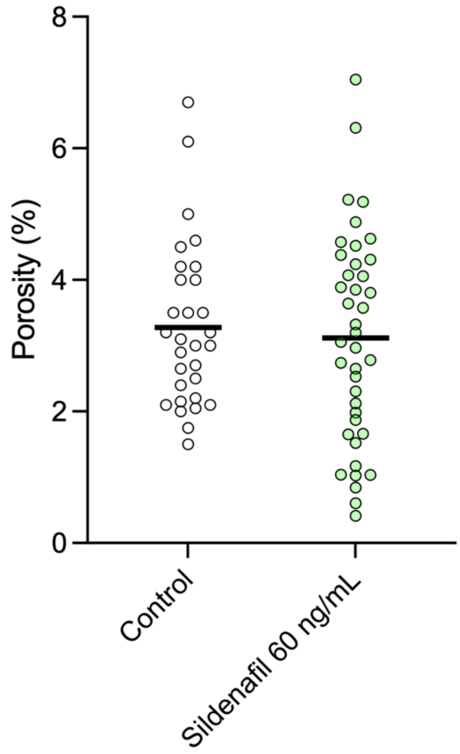

Figure S3. LSEC isolated from aged mice challenged (30 min) with non-encapsulated sildenafil (60 ng/mL) and porosity determined using scanning electron microscopy, n = 2, (unpublished data).

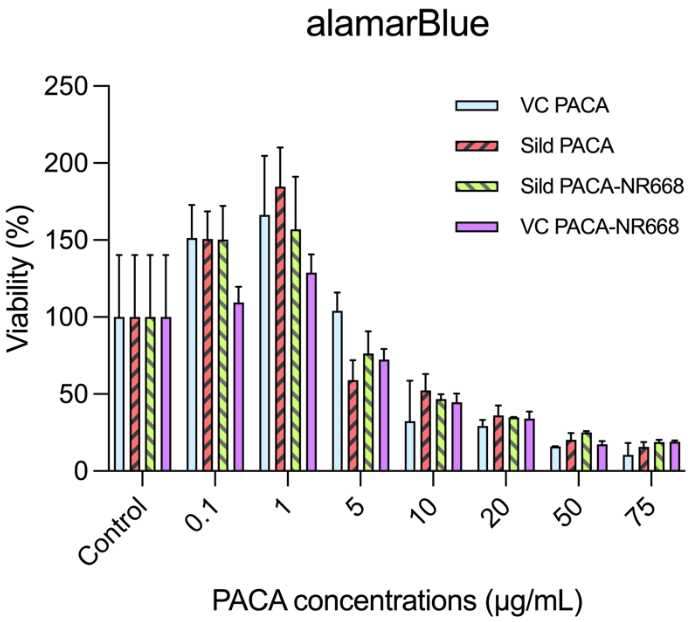

Figure S4. Resazurin assay showing LSEC isolated from aged mice (n = 1) challenged overnight (16 h) with sildenafil PACA (Pill 18), sildenafil PACA-NR668 (Pill 19), vector control PACA (Pill 17), vector control PACA-668 (Pill 20).

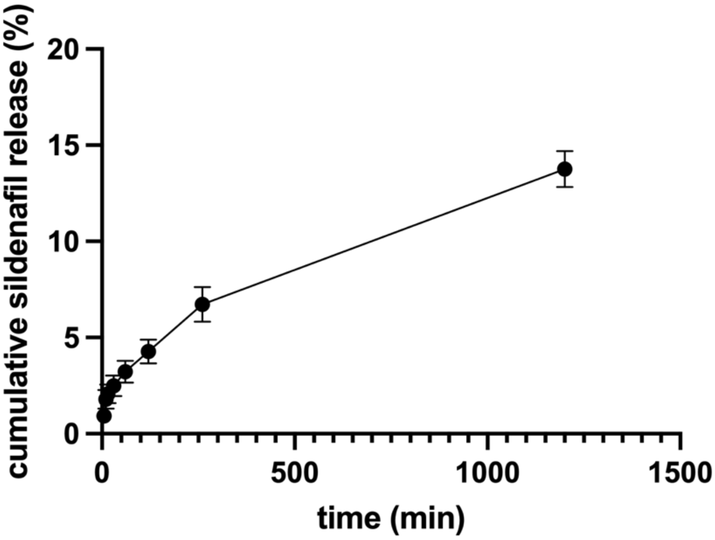

Figure S5. Cumulative release of sildenafil from PACA NPs incubated at 37 ºC in TBS with 0.05% Tween pH 7.6. The datapoints show averages of three replicates.

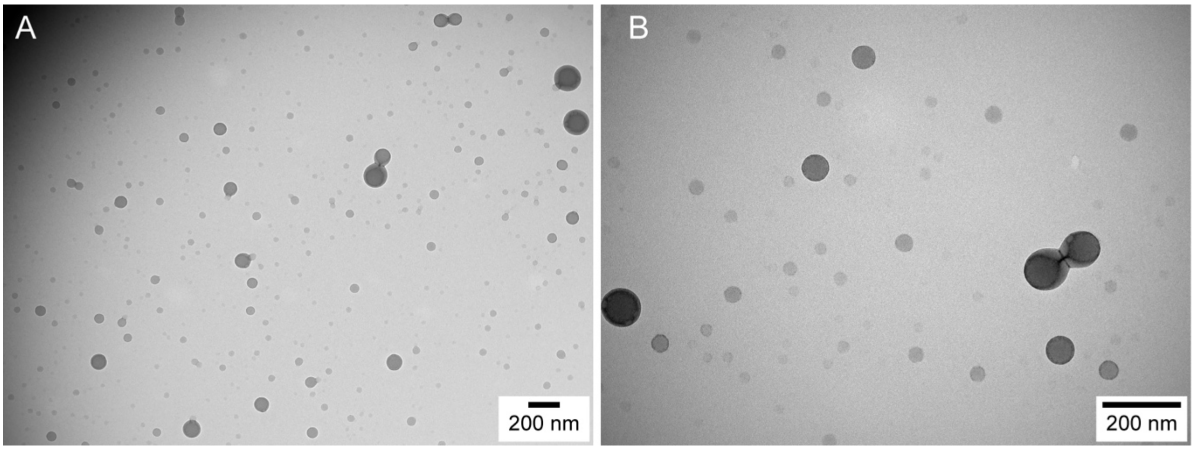

Figure S6. Transmission electron micrographs of PACA nanoparticles. Sildenafil PACA (Pill 81 in Table 1) was diluted 1:100 in double distilled H_2O, and pipetted (5 µL) onto a 400 mesh Cu-grid with carbon coated formvar film. The sample was quickly washed with double distilled H_2O (4–5 drops) followed by incubation with 1% uranyl acetate for 1 min. Excess uranyl acetate was removed using blotting paper and sample then dried prior to imaging on a Hitachi HT7800 120 kV transmission electron microscope.

Reference:

1. Hansen B, Longati P, Elvevold K, Nedredal G-I, Schledzewski K, Olsen R, et al. Stabilin-1 and stabilin-2 are both directed into the early endocytic pathway in hepatic sinusoidal endothelium via interactions with clathrin/AP-2, independent of ligand binding. Exp Cell Res. 2005; 303: 160-73.
